# Clearing techniques for deeper imaging of plants and plant–microbe interactions

**DOI:** 10.1186/s42649-024-00098-9

**Published:** 2024-05-31

**Authors:** Ki Woo Kim

**Affiliations:** Department of Forest Ecology and Protection, Tree Diagnostic Center, Kyungpook National University, Sangju, 37224 Republic of Korea

**Keywords:** Cell wall, Fiber, Light scattering, Pigment

## Abstract

Plant cells are uniquely characterized by exhibiting cell walls, pigments, and phenolic compounds, which can impede microscopic observations by absorbing and scattering light. The concept of clearing was first proposed in the late nineteenth century to address this issue, aiming to render plant specimens transparent using chloral hydrate. Clearing techniques involve chemical procedures that render biological specimens transparent, enabling deep imaging without physical sectioning. Drawing inspiration from clearing techniques for animal specimens, various protocols have been adapted for plant research. These procedures include (i) hydrophobic methods (e.g., Visikol™), (ii) hydrophilic methods (Sca*l*eP and ClearSee), and (iii) hydrogel-based methods (PEA-CLARITY). Initially, clearing techniques for plants were mainly utilized for deep imaging of seeds and leaves of herbaceous plants such as *Arabidopsis thaliana* and rice. Utilizing cell wall-specific fluorescent dyes for plants and fungi, researchers have documented the post-penetration behavior of plant pathogenic fungi within hosts. State-of-the-art plant clearing techniques, coupled with microbe-specific labeling and high-throughput imaging methods, offer the potential to advance the *in planta* characterization of plant microbiomes.

## Introduction

**Fig. 1:**
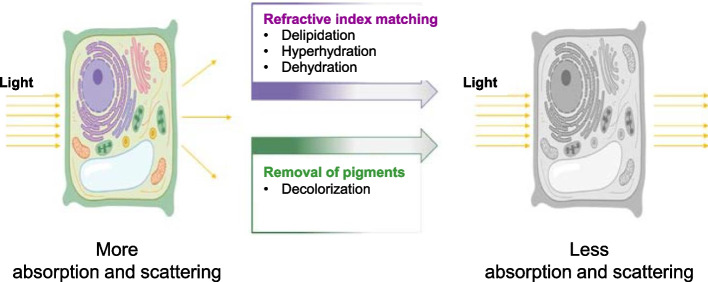
Schematic illustration of optical clearing of a plant cell. Light absorption and scattering within the cell can be reduced through optical clearing. Adapted from Hériché et al. 2022 with permission from the publisher

Clearing techniques encompass a series of chemical procedures designed to render large biological specimens transparent to light, facilitating three-dimensional (3D) deep imaging of large volumes without the need for physical sectioning (Ariel 2017). The microscopic study of biological specimens has long been challenging due to their inherent thickness, opacity, and three-dimensional nature. Conventional histology methods typically involve time-consuming cutting and examination of only a few selected sliced sections of complex 3D organisms (Susaki 2022). However, recent decades have witnessed significant progress in clearing techniques, particularly in animal research (Yamada et al. 2024). Tissue clearing typically involves four main steps: (i) fixation, (ii) permeabilization, (iii) decolorizing, and (iv) RI matching (Fig. 1) (Tainaka et al 2016). The goal of all clearing techniques is to achieve specimen transparency by homogenizing the specimen’s RI through the removal, replacement, or modification of some of its cellular components (Ariel 2017). For instance, a clearing technique may involve the removal of lipids (with an RI of approximately 1.47) and the replacement of intra- and extracellular fluids (RI 1.35) with a solution having an RI equivalent to the remaining protein constituents (RI > 1.50) (Richardson et al. 2021). Clearing techniques have demonstrated the potential to provide more spatially integrated views of the inner workings of organisms (Richardson and Lichtman 2015).

Various clearing techniques originally developed for animal tissues, such as ClearSee, thiodiethanol, PEA-CLARITY, and urea-based methods, have been successfully adapted for use with plant specimens, either with or without modifications (Attuluri et al. 2022). Plant specimens inherently contain cell walls, sclerenchyma fibers, pigments, and phenolic compounds (Hériché et al. 2022). Certain plant tissues present additional challenges due to their dense extracellular molecular mesh, necessitating the use of specialized detergents to enhance tissue permeability (Attuluri et al. 2022). These characteristics contribute to a high degree of inhomogeneity in the RI of the various inter- and intracellular components (Musielak et al. 2016). To address these challenges, numerous approaches employing various reagents and procedures have been developed for clearing plant specimens. Early works of plant clearing were performed using 75% or pure lactic acid by which the vascular structure of flower became transparent (Simpson 1929; Sporne 1948). This review aims to offer guidance for selecting clearing techniques for studies on plants and plant–microbe interactions based on research objectives, imaging modalities, plant species, and specific tissues/organs under investigation. Additionally, representative examples of major clearing techniques, along with brief protocols for each, are provided in chronological order.

## Main text

### Types of plant clearing techniques

The concept of “clearing,” first proposed by Hoyer in the late nineteenth century, aimed to preserve plant specimens using chloral hydrate (Hoyer 1882; Kurihara et al. 2015). Initially, chloral hydrate served as a preserving and mounting medium in Hoyer’s solution (Hériché et al. 2022). Notably, the protocols developed by Lundvall in Sweden and Spalteholz in Germany at the beginning of the twentieth century are considered pioneering works in clearing techniques for animal tissues (Richardson and Lichtman 2015; Lundvall 1905; Spalteholz, 1911).

Clearing techniques are generally classified into two groups based on the physicochemical properties of the clearing agents: (i) organic solvent-based clearing techniques and (ii) aqueous solution-based clearing techniques (Richardson and Lichtman 2015). However, recent reviews on clearing techniques used in animal research have divided these techniques into three categories: (i) hydrophobic methods, (ii) hydrophilic methods, and (iii) hydrogel-based methods (Ueda et al. 2020b; Susaki 2022). Although a recent study proposed categorizing plant clearing techniques into four groups (Hériché et al. 2022), this review will focus on a simple, newly proposed classification for plant systems consisting of three groups (Table 1). Major plant clearing techniques developed since 2010 will be chronologically presented.

**Table 1:** Three major classifications of plant clearing methods and representative examples with clearing agents since 2010

Year	Hydrophobic methods	Hydrophilic methods	Hydrogel-based methods
2013	Visikol™^a^ (Alcohols)		
2014		Sca*l*eP^b^ (Urea and Triton X-100)	
2015		ClearSee^c^ (Urea and xylitol)	PEA-CLARITY^d^ (Sodium dodecyl sulfate)
2016		TOMEI^e^, TDE^f^ (Thiodiethanol)	
2022		iTOMEI^g^ (Caprylyl sulfobetaine and iohexol)	

^a^Villani et al. 2013

^b^Warner et al. 2014

^c^Kurihara et al. 2015

^d^Palmer et al. 2015

^e^Hasegawa et al. 2016

^f^Hasegawa et al. 2016; Musielak et al. 2016

^g^Sakamoto et al. 2022

Most clearing techniques can be used for multiscale imaging of tissues, cells, and organs of diverse plant species through light or fluorescence microscopy. Clearing methods that use water-soluble reagents (hydrophilic methods) are better at preserving the fluorescence of fluorescent proteins and are less toxic compared with approaches that use organic reagents (Ueda et al. 2020a). Although hydrophobic methods usually shrink the tissues and allow the imaging of larger samples, hydrophilic and hydrogel-based methods can expand the specimens, which further increases the transparency and effective resolution (Ueda et al. 2020b). To take full advantages of the clearing techniques, light-sheet fluorescence microscopy became a popular imaging tool for plant volumetric imaging due to its high-speed acquisition of a large field of view (Ovečka et al. 2022; Ueda et al. 2020b).

### Uses of plant clearing techniques

#### Foliar trichomes

**Fig. 2:**
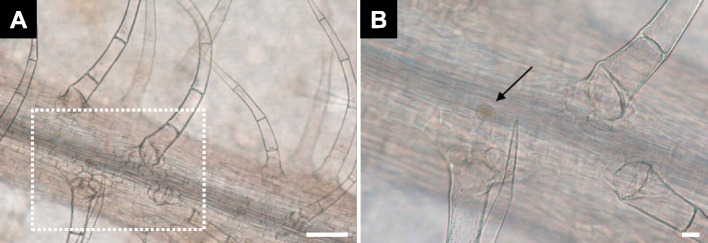
*Origanum vulgare* trichomes cleared with Visikol™. **A** Trichomes with thick cell walls over the vein. **B** Magnified view of the dotted rectangle in (A). The arrow indicates the capitate glandular trichome. Scale bars = 100 μm (A) and 20 μm (B). Adapted from Villani et al. 2013 with permission from the publisher

Oregano (*Origanum vulgare*) is an herb often utilized in anti-inflammatory treatments. In a study by Villani et al. (2013), fresh leaves were immersed in Visikol™ solution until achieving transparency, a process typically lasting 20–30 min. The Visikol™ solution consists of a polychlorinated alcohol mixture for dehydration and glycerol for RI matching (Ueda et al. 2020b). After clearing, the specimens were mounted on a microscope slide glass using one or two drops of Visikol™ solution, after which they were covered with a cover slip. The cleared leaves exhibited non-glandular trichomes over the vein and capitate glandular trichomes (Fig. 2). Originally employed for quality assessment of herbal products, Visikol™ solution (RI 1.4450) has been demonstrated to be an effective clearing agent comparable to acidified chloral hydrate in glycerol (RI 1.4280) (Villani et al. 2013).

#### *Arabidopsis thaliana* seedlings

**Fig. 3:**
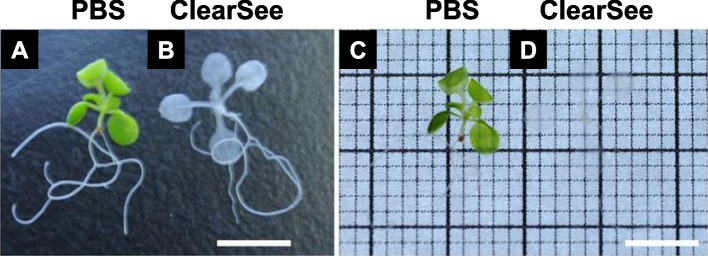
*Arabidopsis thaliana* seedlings. **A** and **C** Fixed seedlings washed in phosphate-buffered saline (PBS). **B** and **D** Fixed seedlings washed in PBS and cleared with ClearSee solution. The seedlings on the right (C and D) are shown on the illuminator. Scale bars = 5 mm. Adapted from Kurihara et al. 2015 with permission from the publisher

*Arabidopsis thaliana*, a small dicotyledonous plant, is widely employed as a model species for plant research. *Arabidopsis* seedlings were fixed with 4% (w/v) paraformaldehyde (PFA) for 30 min, washed in phosphate-buffered saline (PBS), and then subjected to clearing with ClearSee solution at room temperature for 7 days (Kurihara et al. 2015). The ClearSee solution consisted of 10% (w/v) xylitol (for dehydration and RI matching), 15% (w/v) sodium deoxycholate (for delipidation), and 25% (w/v) urea (for hydration) in water (Kurihara et al. 2015; Ueda et al. 2020a, b). In contrast to seedlings washed only in PBS, the ClearSee solution rendered the fixed seedlings transparent (Fig. 3). This clearing regimen proved effective for whole-organ/plant fluorescent imaging (Kurihara et al. 2015).

#### *Nicotiana tabacum* leaves

A hydrogel-based clearing method known as CLARITY (Clear Lipid-exchanged Acrylamide-hybridized Rigid Imaging/Immunostaining/in situ-hybridization-compatible Tissue hYdrogel) was initially developed for animal research (Chung et al. 2013). However, its application to plant research was impeded by the presence of the plant cell wall, which acted as a permeability barrier. To address this challenge, Australian scientists developed Plant-Enzyme-Assisted-CLARITY, or PEA-CLARITY, by incorporating cell wall-degrading enzymes to enhance optical clearing and facilitate antibody probe penetration (Palmer et al. 2015).

Briefly, *Nicotiana tabacum* leaf discs measuring 7 mm in diameter were drop-fixed with 50 ml of ice-cold CLARITY hydrogel solution containing 4% PFA, 4% acrylamide, and other components (Palmer et al. 2015). The fixed specimens were subjected to vacuum infiltration of the hydrogel and polymerized at 37 °C overnight. Subsequently, they were washed in a 50 ml sodium dodecyl sulfate clearing solution for 2 days. Cleared specimens underwent enzyme degradation using an enzyme cocktail mix containing cellulase and amylase at 37 °C for 5 to 7 days. For fluorescent imaging and immunofluorescence, the specimens were stained with Calcofluor White for 20 min and treated with antibodies before enzyme degradation, respectively.

**Fig. 4:**
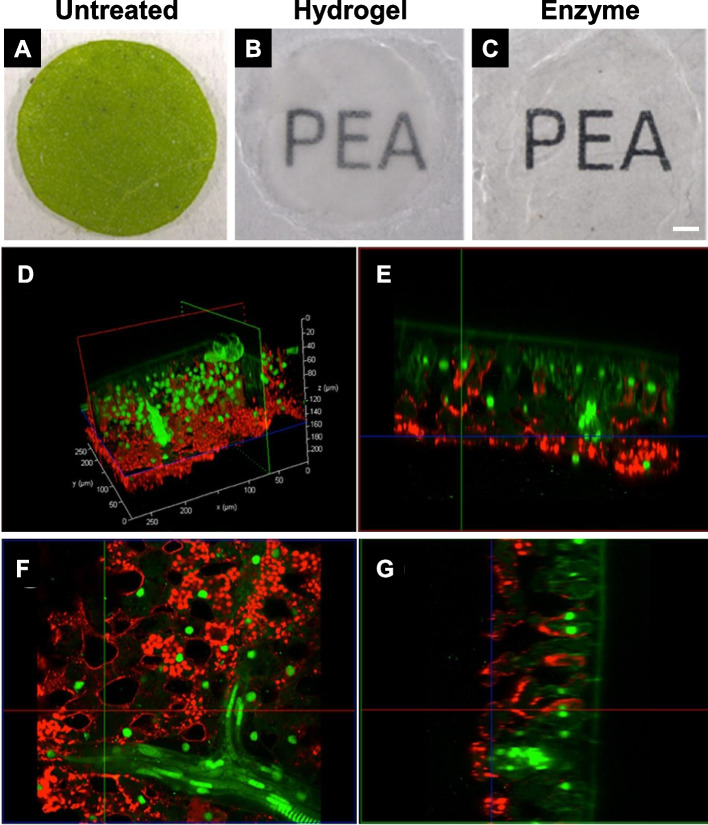
*Nicotiana tabacum* leaves. **A** Untreated leaf disc. **B** Fixed, hydrogel-embedded, and passively cleared leaf disc. **C** Passively cleared and cell wall-degrading enzyme-treated, referred to as PEA-CLARITY treated, leaf disc. Scale bar = 1 mm. **D** to **G** Confocal laser scanning microscope images showing immunostaining of RuBisCO and retention of GFP fluorescence. **D** Three-dimensional projection. **E** to **G** X, Y, and Z slices, respectively. Sv-40 (nuclear-localized GFP) is depicted in green. Tobacco RuBisCO primary and Cy5 secondary antibodies are shown in red. Adapted from Palmer et al. 2015 with permission from the publisher

Upon the removal of lipids, chlorophyll, and other pigments, the leaf discs became optically transparent (Fig. 4A and B) (Palmer et al. 2015). When cell walls and starch were enzymatically degraded, the cleared specimens exhibited greater transparency than those embedded in hydrogel alone (Fig. 4C). Protein retention and antibody penetration were assessed via CLSM (Fig. 4D-G). Immunolabeling of RuBisCO was evident in the chloroplasts of mesophyll cells.

#### *Oryza sativa* leaf blades

Hasegawa et al. (2016) developed a clearing protocol, which they named “Transparent plant Organ MEthod for Imaging” (TOMEI). This technique requires only several hours of fixation and treatment with 2,2'-thiodiethanol (TDE) (RI 1.47), enabling the observation of fluorescent proteins and stains in optically transparent tissues. The terms TOMEI-I and TOMEI-II originate from two different fixative solutions. TOMEI-I is derived from fixative solution I (acetic acid:ethanol = 1:3), whereas TOMEI-II comes from fixative solution II (4% PFA in PBS, pH 7.0).

**Fig. 5:**
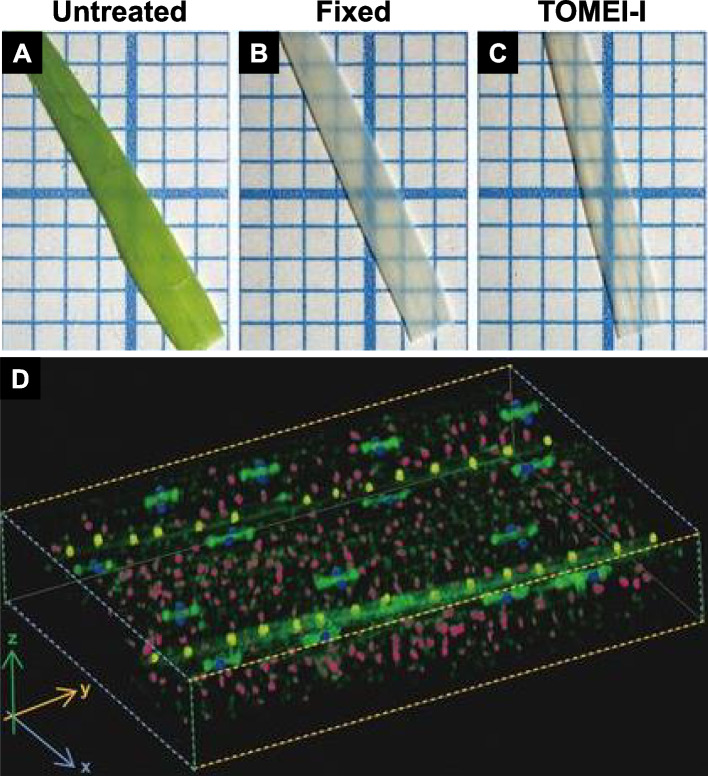
*Oryza sativa* leaf blades. **A** Untreated blade. **B** Fixed blade immersed in solution I (acetic acid:ethanol = 1:3) at 25 °C for 1 h. **C** TOMEI-I cleared blade fixed in solution I and treated with 97% 2,2'-thiodiethanol at 25 °C for 20 min. **D** Three-dimensional image of the fixed, stained, and cleared leaf blade using a confocal laser scanning microscope. The nuclei of guard cells, mesophyll cells, and epidermal cells are colored blue, pink, and yellow, respectively. The green color represents autofluorescence from cell walls. Adapted from Hasegawa et al. 2016 with permission from the publisher

Two-week old rice (*Oryza sativa*) leaf blades (approximately 80 μm thick) were treated with fixative solution I at 25 °C for 1 h. Subsequently, the fixed blades were washed in 70% ethanol and PBS for 10 min each and stained with SYBR Green 1 or 4′,6-diamidino-2-phenylindole in PBS. The stained blades were then treated with 97% TDE at 25 °C for 20 min and observed via CLSM. TOMEI-I revealed no alterations in leaf morphology. However, it rendered the leaves more transparent compared to their untreated and fixed counterparts (Fig. 5A-C). Guard cells, mesophyll cells, and epidermal cells of veins could be clearly distinguished in a 3D image obtained via CLSM (Fig. 5D).

#### *Arabidopsis thaliana* seeds

**Fig. 6:**
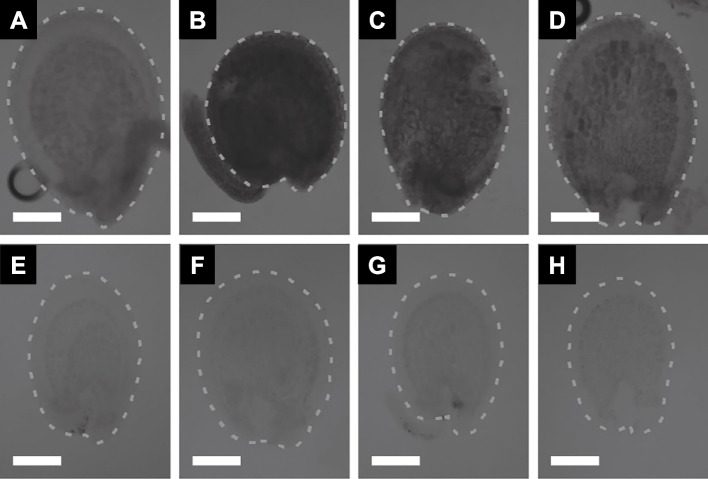
*Arabidopsis thaliana* seeds embedded in different solutions. **A** 10% glycerol, **B** water, (**C**) 20% 2,2'-thiodiethanol (TDE), (**D**) 40% TDE, (**E**) 60% TDE, (**F**) 80% TDE, (**G**) 95% TDE, and (**H**) chloral hydrate. The dotted lines represent the approximate boundaries of the seeds. Scale bars = 50 μm. Adapted from Musielak et al. 2016 with permission from the publisher

Originally used as a clearing agent for animal tissues, TDE was also introduced for plant specimens, where 20% TDE proved effective for detecting fluorescent proteins (Hasegawa et al. 2016). Immature seeds of *A. thaliana* were fixed in 4% PFA at room temperature for 1 h, washed in water, and then transferred to solutions with varying concentrations of TDE at room temperature for 1 h (Musielak et al. 2016). Although the seeds became progressively more transparent in a TDE concentration-dependent manner, no further clearing was observed when the concentrations of TDE reached 60% to 95% (Fig. 6). Compared to ClearSee, which requires several days for incubation, TDE-based clearing can be achieved within a few hours (Musielak et al. 2016).

#### Bryophyte organs

Although TOMEI effectively clears plant specimens, it leaves certain pigments, including chlorophylls, intact, and reduces the fluorescence intensities of fluorescent proteins (Sakamoto et al. 2022). To address these limitations, an improved version of the TOMEI method, referred to as iTOMEI, was developed. iTOMEI incorporates caprylyl sulfobetaine for decolorization, a weak alkaline solution for enhanced fluorescence signal, and an iohexol solution for achieving a higher RI.

*Marchantia polymorpha*, widely known as the common or umbrella liverwort, is widely distributed worldwide. In a recent study, its organs were fixed in 1% PFA in PBS for 1 h and washed three times in PBS for 5 min each (Sakamoto et al. 2022). Subsequently, they were treated with a decolorization solution (100 mM sodium phosphate buffer at pH 8.0 with 20% (w/v) caprylyl sulfobetaine) for 24 h. After washing in PBS, the decolorized specimens were stained with Calcofluor White for 10 min and incubated in 70.4% (w/v) iohexol in PBS for 1 h. Finally, the samples were mounted on a microscope slide glass with 70.4% (w/v) iohexol.

**Fig. 7:**
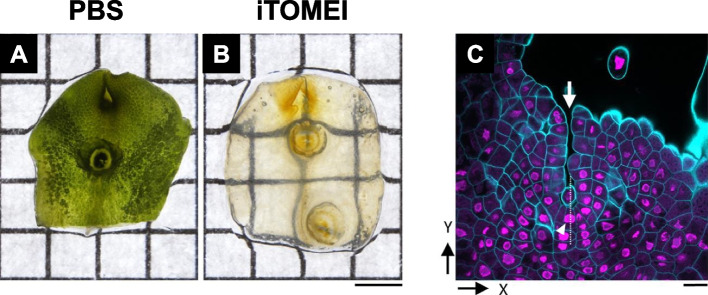
*Marchantia polymorpha* thalli. **A** Fixed thallus washed in PBS. **B** Fixed thallus washed in PBS and cleared with iTOMEI. Scale bar = 2 mm. **C** Confocal laser scanning micrograph of a fixed thallus expressing histone H2B-tdTomato (magenta) cleared with iTOMEI and stained with Calcofluor White (cyan). The arrow and arrowhead indicate an apical notch and a fan-shaped cell, respectively. Scale bar = 50 μm. Adapted from Sakamoto et al. 2022 with permission from the publisher

An apical portion of the thallus treated with iTOMEI was compared with a sample washed in PBS (Fig. 7A and B). With fluorescent proteins being clearly visible, both apical and subapical cells were detected using CLSM (Fig. 7C).

#### Wheat-fungal pathosystem

**Fig. 8:**
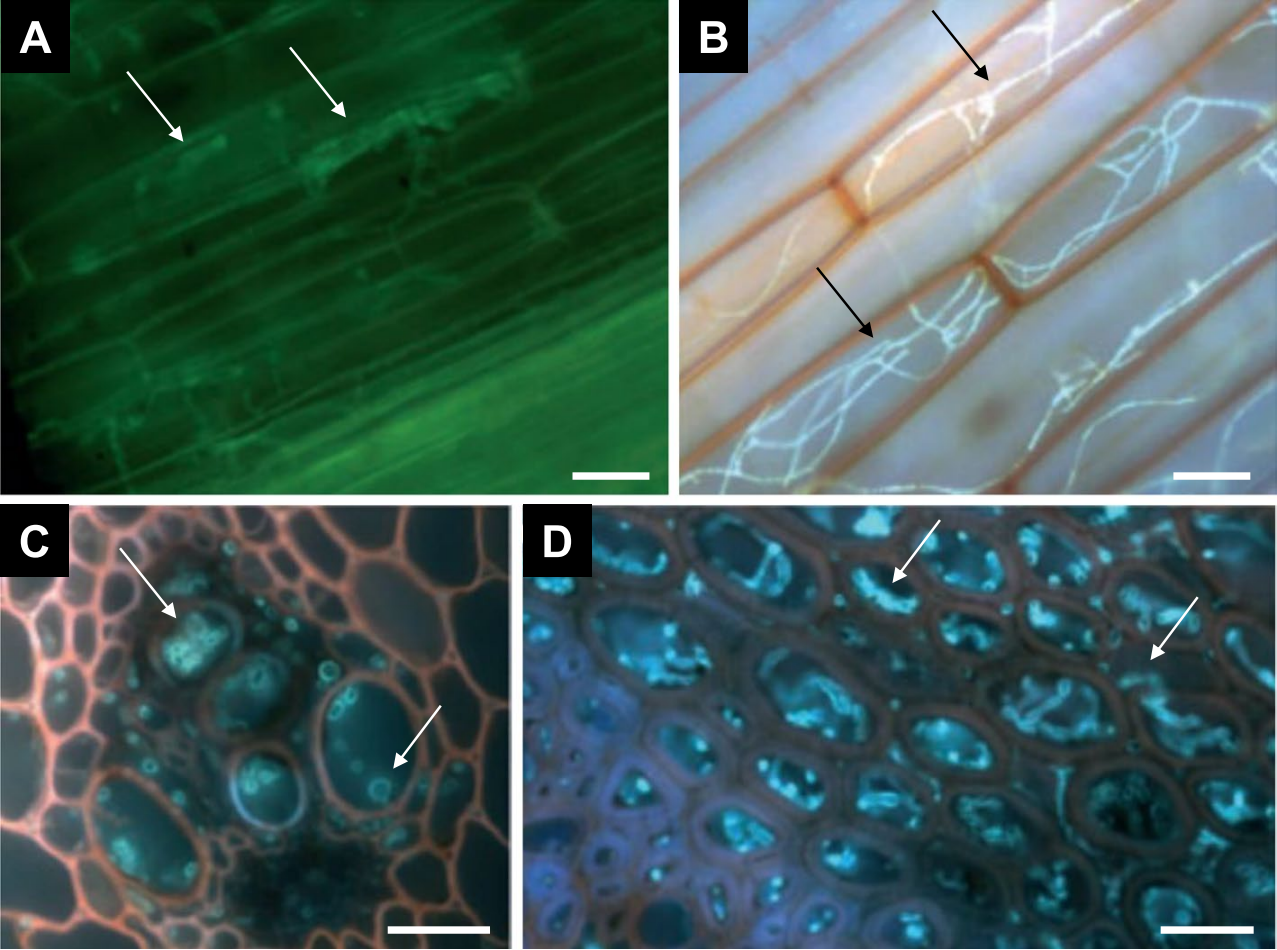
Fluorescence micrographs of bread barley leaves. **A** and **B** Longitudinal sections. **A** Fixed, cleared, and stained leaf with solophenyl flavine 7GFE only. **B** Fixed, cleared, and stained leaf with safranin and solophenyl flavine 7GFE. *Puccinia striiformis* hyphae are visibly distinct from the host tissues. **C** and **D** Transverse sections of uncleared and stained leaves with safranin and solophenyl flavine 7GFE. **C** *Fusarium pseudograminearum* hyphae (cyan) growing within the vascular tissue (red) of a wheat internode. **D** *Fusarium pseudograminearum* hyphae (cyan) growing within the non-vascular nodal tissue (purple). The arrows indicate hyphae. Scale bars = 25 μm (**A** and **B**) and 50 μm (**C** and **D**). From Knight and Sutherland 2011 with permission from the publisher

Bread wheat (*Triticum aestivum*) leaves infected with *Puccinia striiformis* or *Fusarium pseudograminearum* were immersed in a clearing solution containing 0.15% (w/v) trichloroacetic acid in ethanol:chloroform (4:1; v/v) for 48 h (Knight and Sutherland 2011). The leaves were then successively washed in 50% ethanol, 50 mM NaOH, and MilliQ water, followed by incubation in 0.1 M Tris/HCl (pH 8.5). Afterward, the leaf samples were stained with safranin (for plant cell wall) or solophenyl flavine 7GFE (for fungal cell wall). Alternatively, some leaves were stained and observed using fluorescence microscopy without clearing. Optimal images were obtained after staining tissues with both dyes (Fig. 8A and B). Transverse sections revealed hyphal growth in the intercellular space as well as within host cells without prior clearing (Fig. 8C and D). This clearing solution has previously been employed to observe powdery mildew growth in barley leaves (Wolf and Frič, 1981). Additionally, conidial germination and appressorium formation of *Botryosphaeia dothidea* were observed on apple peels using the same clearing solution (Kim et al. 2005).

#### Maize-fungal pathosystem

**Fig. 9:**
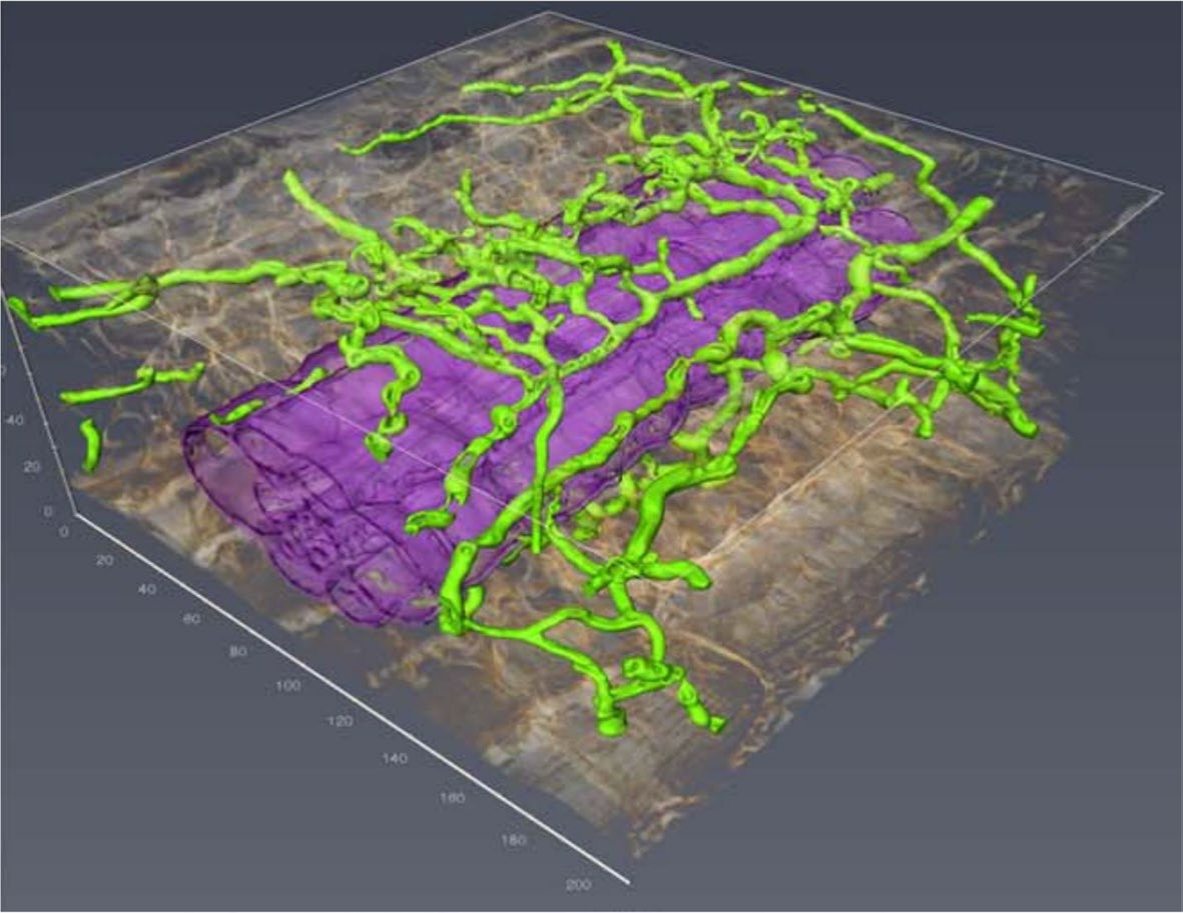
Confocal laser scanning micrograph of *Cochliobolus heterostrophus* in a fixed and cleared maize leaf. Fungal hyphae (green) are observed around the leaf vascular bundle (purple). Adapted from Minker et al. 2018 with permission from the publisher

Maize (*Zea mays*) leaves were inoculated with a spore suspension of *Cochliobolus heterostrophus* (Minker et al. 2018). Briefly, the leaves were fixed with a solution containing 2% (v/v) glutaraldehyde, 2% (w/v) PFA, and 0.05% (v/v) Triton X-100. Subsequently, they were briefly incubated in 0.2 M glycine and stained with Calcofluor White MR2 and wheat germ agglutinin conjugated with Alexa Fluor 568 for 5 days. Following staining, the leaves were transferred to Sca*l*eP solution containing 6 M urea, 30% (v/v) glycerol, and 0.1% (v/v) Triton X-100 in sterile water for 48 h (Warner et al. 2014). Sca*l*e, originally developed for clearing mammalian tissues, was modified for use with plant specimens. CLSM imaging revealed fungal hyphae around the vascular bundle of the fixed and cleared maize leaf (Fig. 9) (Minker et al. 2018).

## Conclusions and outlook

Most clearing techniques for plants have been developed and extensively utilized for deep imaging of herbaceous plants, including major food crops and well-established model plants such as *Arabidopsis thaliana* and *Nicotiana tabacum*. Recently, clearing techniques for woody plants have also been reported across diverse woody plant taxa (Xia et al. 2021; Jele et al. 2023). The applications of cleared, transparent wood in architecture encompass various functionalities, including intelligent windows, lighting equipment, solar cell light management, aesthetic decoration, and other potential functions (Yang et al. 2023). In terms of the histo- and cytopathology of trees, shrubs, and vines, delignified transparent large woody organs are well-suited for in situ imaging of wood-decaying fungal behavior within secondary wood tissues. Moreover, *in planta* localizations of plant microbiomes and holobiont components could be significantly enhanced through the integration of state-of-the-art plant clearing strategies, compatible microbe-specific labeling, and high-throughput imaging techniques.

## Data Availability

Data and materials are available on request.

## Abbreviations

CLARITY: Clear Lipid-exchanged Acrylamide-hybridized Rigid Imaging / Immunostaining / in situ-hybridization-compatible Tissue hYdrogel
CLSM: Confocal laser scanning microscope
GFP: Green fluorescent protein
PBS: Phosphate-buffered saline
PEA-CLARITY: Plant-Enzyme-Assisted (PEA)-CLARITY
PFA: Paraformaldehyde
RI: Refractive index
RuBisCO: Ribulose bisphosphate carboxylase
TDE: 2,2'-Thiodiethanol
TOMEI: Transparent plant organ method for imaging
